# Bulletin of the Buffalo General Hospital

**Published:** 1911-10

**Authors:** 


Bulletin of the Buffalo General Hospital

VOL. I OCTOBER, 1911 No. 2

### The Harrington Hospital

THE Harrington Hospital for Children, a department of The Buffalo General Hospital, was opened for the reception of patients October 1st. This building is a gift of the late Dr. Devillo W. Harrington, and, as its name signifies, is to be used for children. It is absolutely fireproof in construction, and as the building has been finished for some time, there has been ample opportunity for testing every detail before placing patients in it. The ventilation and heating, after thorough trial, have proven most satisfactory. A food carrier, built on the principle of the fireless cooker, will be used as a conveyor of foods from the main kitchen to the service rooms. This carrier, after a careful test, demonstrated that a meal could be kept hot in it for two hours, should such a demand be made.

The hospital has four wards of twelve beds each, eight private rooms, two rooms for infants whose mothers will occupy nearby rooms or wards, and two operating rooms, besides blanket warming rooms, dining rooms, service rooms, ample bath- and toilet-rooms, lockers, store rooms, and the like. There are also large detention rooms for the reception of patients, where children whose diagnosis is not perfectly clear will be isolated till a positive one can be made. The hospital has six porches—two facing south, two east, and two west—of sufficient area to accommodate at one time one-half the total number of patients which the building can receive.

One of the important departments of the work will be the maternity service, which will be installed on the second floor of the hospital. On this floor there is a special delivery room with all conveniences, including equipment for obstetric surgery, private rooms, the rooms especially for infants, and ample ward space. The General Hospital during the past year has had a large maternity service, due to the establishment of several free beds by friends of the hospital, and with these increased facilities and the skilled care of Dr. Van Peyma and his assistants, there is every reason to believe that this department of the hospital work will become a very important one.

The first floor is devoted to the larger children. Here there is a second operating room, especially equipped for such patients. In addition to the sunny wards, private rooms and full service equipment devoted to them, a playroom, 24 x 50 feet, has been provided. This is to be supplied with sand boxes, games, and other such therapeutic agents; it is in reality an indoor playground, to supplement the larger outdoor grounds in inclement weather. These latter are in the rear of the building, and give ample space for out-door amusements and exercise.

The opening of the Harrington Hospital is in every way a cause for congratulation. It sets free space in the General Hospital which is much needed for other purposes, insures greater quiet in that building, and gives the children and the maternity cases greater privacy and a fuller consideration of their needs than is possible as part of a general service.

---

### The Enlargement of the Operating Room

THERE is no better indication of the way in which the hospital keeps pace with the advance in medicine than the improved facilities for operative surgery. Some members of the staff can remember when there was but one operating room, and that imperfectly equipped. The last addition gives us six operating rooms, each well prepared for service.

The amphitheatre is, of course, the largest and most complete, being built of marble and white tile, and equipped with practically every appliance required for general surgery. Adjoining this and of the same construction, there was, in the original arrangement, a smaller operating room, a room for anaesthetising, one for disinfection of the hands, one for the sterilisers, one for supplies and a fifth for instruments. It often happened, however, that when both of these operating rooms were in use a third in that immediate vicinity was desired. Moreover, advancing science has shown that good results depend much upon having as complete disinfection as possible of not only the surgeon's hands, but his entire body. Therefore, by the wise action of the trustees, a small ward and one adjoining room have been added to the operating equipment. Each surgeon has now a locker in which to hang the clothes ordinarily worn, in an adjoining compartment he can have a shower bath, and then, donning a suit of sterilised clothing, can enter the operating room practically free from germ-carrying material. There is also an apartment in which the nurses can make such changes in their dress as may be necessary. This extension has made it possible to change the former supply room into an operating room, thus giving three such rooms in that group. There is also a special gynecological operating room in connection with the women's wards. With the two now available in the Harrington, the General Hospital puts six well equipped operating rooms at the service of the medical profession.

The house formerly occupied by Dr. Ross has now been removed, and the frame building just west of it, which is owned by the hospital, is also to be taken away. These changes add about 7,800 square feet to the open grounds of the institution. Plans for their improvement have already been made.

---

The smoke consuming device installed in the power house last year has not only eliminated the smoke nuisance, so far as the hospital chimney is concerned, but in ten months effected a saving of 428 tons of coal.

---

Mr. Pardee, president of the Board of Trustees, and Dr. Ross, attended the meeting of the American Hospital Association, which convened in New York on September 19th. The Association, whose purpose is to promote economy and efficiency in hospital management, presented a strong program. Dr. Ross opened the discussion of "Hospital Annual Reports; What for? Who for?" a paper to be given by Mr. Parke, of the Montreal General Hospital.

---

### The Semi-private Rooms

THE opening of the Harrington Hospital makes possible a very important addition to the resources of the General Hospital itself. The wards vacated by the transfer of the children and the maternity cases will be transformed into semi-private rooms, making, with those already in use, twenty-seven in all. The three semi-circular wards, which are exceptionally pleasant, and have full south exposure, are to be devoted to this service. Each ward is divided by heavy wooden screens into nine sections, giving on the central space where the nurse in charge has her desk. These screened-off spaces accommodate comfortably the furniture of a small room, are bright and sunny, and have ventilation of an excellence which a completely shut-off space of that size rarely possesses. The baths and toilet rooms in connection with these rooms are ample, as they were planned for a ward service accommodating a much larger number of patients. Each group of rooms has immediately adjacent a pantry with hot tables and other appliances for getting the patients' meals to them in appetising condition. The charge for these rooms is two dollars a day, with corridor nursing—that is, the service of a nurse who has charge of four patients.

The extension of this service is significant because it meets the needs of a class who have hitherto not found hospital care readily available. The desires of the well-to-do have been carefully considered; the poor need never lack hospital care; but there has been but scanty provision for persons of moderate income. These semi-private rooms are offered with the confidence that they meet a social need.

---

### Report of the State Board of Charities

THE following extracts from the last report of the inspector of the State Board of Charities give a good idea of some phases of the hospital's internal economy:

"The hospital is in charge of a superintendent who is a physician. He is assisted by sixty-five women and twenty-two men who are general employes. A superintendent of nurses, two assistant superintendents, a night supervisor, one graduate head nurse, three graduate operating room nurses, two dietitians, a paid pathologist, ten internes and thirteen orderlies are also employed. Eighty-two nurses are in training. During the day thirty-five nurses and six orderlies care for ward patients, and eight nurses and two orderlies care for private patients." (This, of course, does not include special private nurses.) "A night engineer, a night supervisor of nurses, twelve nurses, four orderlies and a night clerk are on duty from 7 p. m. to 7 a. m. The house physicians and surgeons make formal rounds morning and evening, and also accompany the attending physicians and surgeons when they make their afternoon rounds. The superintendent of nurses and her assistants visit the patients each day, and two social welfare workers look after special cases that are reported to them by the physicians. The food qualities are good and the food is carefully prepared. The housekeeper, who is a registered nurse and a trained dietitian, is in charge of the main diet kitchen and the private patients' food, while another dietitian is in charge of the main kitchen and the diets for ward patients, nurses and internes.

The general sanitary conditions are good. The beds are comfortable, clean, and well made. They are carbolized and the mattresses, rubber sheeting and pillows are carbolised or steam sterilised and aired in the sun after each patient. Clothing of patients is listed, sterilised if necessary, and put in a general locker room. Soiled clothes are kept in canvas bags in the service rooms and sent to the laundry three times a week. The bags are washed once a week. Dressings are kept in covered metal cans in the service rooms and sent to the crematory two or three times a day, and the small covered garbage pails in the pantries are emptied three times a day in the general garbage receptacle, from which it is removed daily. The ward floors are swept three times daily with brooms with dampened covers on them. The general wards are large airy rooms with windows on three sides. In addition, a fan system of ventilation is in use during the winter months. Admission baths are given to all, and the ward patients have three baths a week.

Admission slips containing all necessary information for vital statistics are made out for each case, and a general record book is kept which contains also additional information. Every case is numbered so that one index answers for all records. A book is kept for the ambulance calls. The patients' charts and medical records are filed in envelopes and kept as permanent records."

---

THE TECHNIQUE OF URETHRAL EXAMINATIONS.—Walter S. Reynolds, New York, believes that when dealing with a chronic urethritis one should make a clear diagnosis of the extent of the disease in order that the treatment may be effective. The history should be carefully inquired into as to duration of the attack, severity, frequency of urination, and as to what time of the day the symptoms are most pronounced. The examination should be made some five or six hours after urination, preferably after a night without it. A loopful of the discharge should be collected for a smear. The anterior urethra should be washed out with a mild boracic acid solution until the fluid returns clear. Urine should be passed into a clean glass which will contain that from the posterior urethra. Prostate and seminal vesicles should be examined after gentle massage, and the rest of the urine passed for examination of the prostatic secretion. Each specimen should be separately examined, and each will reveal the condition of the respective portion of the urethra. Examination for stricture is then to be made.—*Medical Record,* July 1, 1911.

THE RADICAL FRONTAL SINUS OPERATIONS.—John E. MacKenty and Gerhard H. Cocks, New York, describe their method of operation for frontal sinus disease, and give three illustrative cases. They claim for their method that it is simply a combination of the best methods, gives good drainage, and cures the disease as well as Killian's operation, but without deformity. The entire front wall of the sinus should be removed only for necrosis of the bones. The advantages over Killian's operation are avoidance of facial deformity; dislodgement of the superior oblique tendon to facilitate the removal of the floor of the sinus; and adptability of this method in cases which have perforated into the orbit with the production of orbital cellulitis. Diplopia is only transitory.—*Medical Record,* July 1, 1911.

